# Holistic management of DSD

**DOI:** 10.1016/j.beem.2010.01.006

**Published:** 2010-04

**Authors:** Caroline E. Brain, Sarah M. Creighton, Imran Mushtaq, Polly A. Carmichael, Angela Barnicoat, John W. Honour, Victor Larcher, John C. Achermann

**Affiliations:** a Department of Endocrinology, Great Ormond Street Hospital NHS Trust, London WC1N 3JN, UK; b Department of Women's Health, University College Hospital, London NW1 2PG, UK; c Department of Urology, Great Ormond Street Hospital NHS Trust, London WC1N 3JN, UK; d Department of Psychology, Great Ormond Street Hospital NHS Trust, London WC1N 3JN, UK; e Department of Clinical Genetics, Great Ormond Street Hospital NHS Trust, London WC1N 3JN, UK; f Clinical Biochemistry, University College London Hospital, 60 Whitfield Street, London W1T 4EU, UK; g Department of Adolescent Medicine, Great Ormond Street Hospital NHS Trust, London WC1N 3JN, UK; h Developmental Endocrinology Research Group, Clinical & Molecular Genetics Unit, UCL Institute of Child Health, 30 Guilford Street, London WC1N 1EH, UK

**Keywords:** disorder of sex development (DSD), intersex, multidisciplinary team, congenital adrenal hyperplasia, gonadal dysgenesis, ambiguous genitalia

## Abstract

Disorder of sex development (DSD) presents a unique challenge, both diagnostically and in terms of acute and longer-term management. These are relatively rare conditions usually requiring a multidisciplinary approach from the outset and the involvement of a tertiary centre for assessment and management recommendations. This article describes the structure of the multidisciplinary team (MDT) at our centre, with contributions from key members of the team regarding their individual roles. The focus is on the newborn referred for assessment of ambiguous genitalia, rather than on individuals who present in the adolescent period or at other times, although the same MDT involvement is likely to be required. The approach to the initial assessment and management is discussed and the subsequent diagnosis and follow-up presented, with emphasis on the importance of careful transition and long-term support.

The management of a newborn or newly presenting adolescent with a disorder of sex development (DSD) can be a difficult challenge for health-care professionals and one which requires a holistic approach from the outset.

The primary aim should be to achieve a diagnosis, sex assignment (if needed) and management plan as quickly as possible, but without rash decisions being made. Thus, early liaison and assessment by an experienced multidisciplinary team (MDT, or interdisciplinary team) is essential.^1,2^

Ideally, the MDT would be composed of an endocrinologist, urologist, gynaecologist, psychologist, biochemist and clinical/molecular geneticists, with access to ethical advice and with supporting specialist pathology and imaging where needed (Fig. 1). However, DSD can present to many different health-care professionals and at different ages (Table 1). Therefore, an awareness of these potential diagnoses, appropriate terminology and the need to involve specialist services early is required by all practitioners dealing with children and young people.^3–5^

In this article, we outline our experience from the MDT at Great Ormond Street Hospital and detail the various specialists’ input to the overall service. Transition is made to the Adolescent MDT at University College London Hospitals (UCLH), which comprises a similar spectrum of professionals. Thereafter, there is a final transition to the MDT adult DSD service, also at UCLH. Of course, the exact composition of the team may vary from one centre to another and not all the resources described are always available within our setting.

## Presentation of DSD

As noted above, DSD can present to many different health-care professionals and at several different ages (Table 1). Whilst the majority of infants with DSD present with genital ambiguity at or shortly after birth, an increasing number of individuals are identified either antenatally or during childhood and adolescence. Adults diagnosed with DSD will sometimes present through infertility clinics.

### Antenatal

The relatively frequent practice of chorionic villus sampling or amniocentesis for trisomy screening in some countries may identify a mosaic karyotype (e.g., 45,X/46,XY), or the phenotypic sex of the baby on ultrasound or at birth may be found to be discordant with the karyotype (e.g., complete androgen insensitivity syndrome, CAIS). When karyotype–phenotype discordance is present, appropriate education and counselling can be provided to the parents so they are aware of the likely investigations and decisions that may be necessary at birth. In the future, focussed trisomy screening or earlier non-invasive testing may reduce the frequency of this presentation but, by contrast, non-invasive foetal ‘sex determination’ will become easier.^6^ Of some concern is the practice of sexing the foetus during routine antenatal ultrasound scans. This has led to some very difficult situations, particularly when the baby is born unexpectedly with genitalia that are ambiguous or are discordant with the parents' expectations.

### Newborn

Genital ambiguity is the most frequent presentation of DSD in the newborn period. However, it is sometimes masked by local swelling (breech presentation) or may be apparent (e.g., clitoral hypertrophy in the premature infant with little suprapubic fat). The empty scrotum of a genetic (46,XX) female with a severe virilising form of congenital adrenal hyperplasia (CAH) is something which, if missed on the postnatal inspection, can have significant consequences such as adrenal crisis or incorrect sex of rearing.

Bilateral undescended testes should always be referred for further assessment as should the presence of inguinal herniae. Hypospadias occurs in approximately 1:250 newborn boys and will usually be referred to a paediatric urologist. In some cases, more detailed investigation will be required, especially if the form of hypospadias is severe (e.g., penoscrotal) or associated with a small penis with poor corporal consistency and/or undescended testes.

### Childhood and adolescence

Some conditions may present with androgenisation during childhood (e.g., 11β-hydroxylase deficiency) or at adolescence (e.g., 17β-hydroxysteroid dehydrogenase deficiency type 3; 5α-reductase deficiency type 2). Failure to develop in puberty or to achieve menarche may be the first manifestation of some other forms of DSD (e.g., 17α-hydroxylase deficiency, CAIS or gonadal dysgenesis).

## Initial approach to the newborn with DSD

This section focusses on the newborn with genital ambiguity, as this is a relative emergency that requires prompt and efficient communication between the referring hospital and the tertiary centre.

### Management at birth

It is of vital importance that all professionals involved with the management of the newborn within the labour ward and on the postnatal wards are knowledgeable about what to do when a baby is born with indeterminate sex. If in any doubt, the sex of a baby should never be guessed. A senior paediatrician should counsel the parents in the first instance, and a clear plan of further management outlined. A prompt referral to a specialist centre should then be made.

Often, a child with ambiguous genitalia is born at a local hospital, so initial specialist management is at a distance. The presence of ambiguous genitalia is not a medical emergency in itself, so parent–infant interaction is essential and the child should not be unduly ‘medicalised’. However, the adrenal insufficiency associated with some forms of DSD can become an emergency if overlooked and not treated early, so vigilance (e.g., hypoglycaemia) and monitoring (e.g., electrolytes) are necessary until adrenal dysfunction is excluded and the karyotype is known. Steroid and salt replacement treatment will be needed if adrenal insufficiency is suspected.^7^

Information should be given to the family in a consistent manner that is appropriate for their level of understanding, with awareness of religious and cultural beliefs and preconceptions (e.g., their desire for a specific family structure or information about the foetal sex given from prenatal scans).^8^ Parents often experience extreme stress about the disclosure of the problem to family and friends and find uncertainty very unsettling. This issue is compounded if they have already told relatives about the expected sex, or have prepared the baby's room or bought clothes accordingly. Indeed, many neonatal nurseries focus on ‘pink’ or ‘blue’, so even the hospital environment can be extremely challenging.

In addition to the endocrine issues, it is essential to appropriately address social, psychological and urological aspects of management in the early period, and early contact with the MDT can give reassurance to the family whilst also focussing on the appropriate investigations needed to make a diagnosis and in some cases to establish the appropriate sex assignment.

Sending the appropriate samples for rapid karyotype analysis by fluorescence *in situ* hybridisation (FISH) or quantitative fluorescence polymerase chain reaction (QFPCR), and discussing the issues with the relevant clinical and molecular genetics services are extremely useful so that a broad diagnostic category can be reached quickly (see the article on classification this issue).^1–3^

### Initial specialist assessment

Once the referral to the specialist MDT has been made, the initial assessment of the baby is planned on an individual basis, but based on a standard structure of investigations and assessments^9^ (UKDSD Taskforce Guidelines in preparation). Investigating cases of karyotype–phenotype discordance involves a similar approach to that for the newborn with ambiguous genitalia, but the parents are usually more aware of the potential issues if they have been counselled antenatally. Great care must be taken not to instil any preconceptions as to the sex of rearing until the baby has been assessed after birth, as this can result in inappropriate decisions being made with challenging long-term consequences.

If the sex of rearing is unclear, the family will be referred to the specialist centre within a few days. An emergency slot is available where the on-call urologist, endocrinologist and, often, clinical psychologist will assess the baby. There will be a key member of each team who will communicate with individual families, establishing a rapport, ensuring consistent information is given and acting as a point of contact.

It may not be possible to decide on the appropriate sex of rearing at the first meeting as this may depend on the results of biochemical investigations and sometimes also on endoscopic assessment. Most importantly, however, the family should feel supported, and strategies for coping with the uncertainty of gender assignment will be discussed with them. In particular, advice as to how best to impart information to family and friends will be given, which helps to alleviate some of the stress that the family inevitably feels that they are under.

### Additional investigations

An indicator of karyotype, such as rapid FISH analysis, will usually be known by the time of the first meeting, and this together with the genital appearances will guide the subsequent assessment.

The exclusion of any associated adrenal insufficiency is of paramount importance and will be made in almost every case in our centre. A urinary steroid profile ideally forms part of the routine assessment in many cases, as this will not only help with adrenal insufficiency, but will also provide diagnostic information after the third day of life in most forms of CAH, and from 2–3 months for 5α-reductase deficiency.

Whether to stimulate potential testicular tissue with a 3-day or 3-week human chorionic gonadotropin (hCG) stimulation test can be a difficult decision, especially if a female sex of rearing is being considered. The effects of postnatal androgen exposure to the developing brain are unknown, especially if the baby is subsequently raised female. Measurements of anti-Müllerian hormone and inhibin B are proving increasingly useful for assessing gonadal (testicular) integrity, but these assays are not established in all centres and results are not always available quickly if assays are run intermittently or if samples are sent out to specialised centres for analysis.

Genital photography will often be undertaken at the first meeting and again after any gonadal stimulation. However, detailed notes of the genital examination should be made by the urologist as photographs can be misleading (e.g., suprapubic fat pad) and corporal consistency on palpation is not always reflected well by images. Any potential dysmorphic or associated features should be noted and the experience of a clinical geneticist obtained early in such situations, as such signs can be subtle in the early newborn period.

Imaging (e.g., pelvic ultrasound) has a role in locating gonads and possibly identifying Müllerian structures but should be viewed with extreme caution, especially if undertaken by an inexperienced radiologist. This can be misleading, even in experienced hands. The presence of Müllerian structures or descended gonads by themselves should not be the deciding factor in gender assignment, but parents often focus on this information, especially if the limitations of this analysis are not explained.

The aims of the initial assessment and subsequent presentation at the MDT are to establish the appropriate sex of rearing, form a plan of diagnostic procedures and provide psychological support for each individual family as a whole. Several factors need to be considered when counselling the family about gender assignment, such as likely gender identity; urological and sexual function; the likely need for endocrine replacement; fertility options and long-term tumour risk. These factors can be influenced by the underlying diagnosis; therefore, attempts at achieving a specific diagnosis as soon as possible are important. However, it is also extremely important that every case is viewed individually, as the range of phenotypes can vary significantly even within patients with the same underlying diagnosis, and long-term outcome data for many forms of DSD are not readily available.^10^

Of course, it is of vital importance that parents are involved in the decision-making process and are fully aware of the potential implications for the child. To have to contemplate gender reassignment at a later stage is one of the most difficult areas and can take several lengthy consultations between family and professionals. Adopting an MDT approach can help facilitate parents' understanding of the issues, and provides continuity of care and consistency of information. Clear communication between members of the MDT is therefore essential.

## The multidisciplinary team

**Fig. 1:**
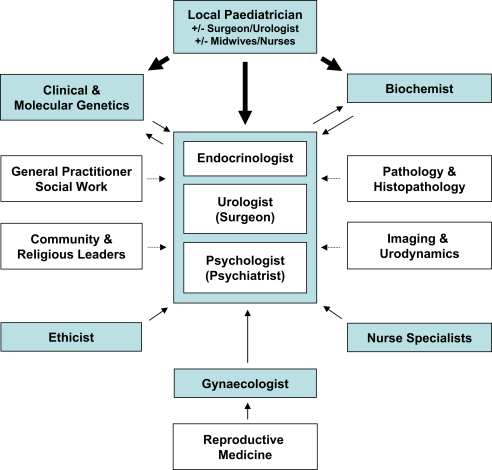
An overview of the multidisciplinary team.

As recommended in the ‘consensus statement’, children with DSD should be managed by an MDT with appropriate expertise and experience in managing these conditions.^1,2^ The exact composition of the team will vary from one centre to the next, but some of the key team members and related disciplines are shown in Fig. 1. Not every professional will be relevant to each individual patient, but it is important that they have the opportunity to express an opinion and provide suggestions.

### An endocrinologist's view

The endocrinologist plays a key role in coordinating investigations and management, and is usually the first point of contact for referral. All endocrinology consultants in our centre are well experienced in the range of disorders that can present with ambiguous or discordant genitalia and are appropriately placed to deal with essential aspects of monitoring (e.g., electrolytes); obtaining and interpreting first-line investigations (e.g., basal and stimulated steroid tests and urine steroid analysis); and coordinating steroid replacement therapy (e.g., glucocorticoids, mineralocorticoids and salt), where necessary. The fact that adrenal disorders associated with DSD are common and are a medical emergency if not recognised and treated appropriately means that the endocrine team is central to the initial stages of assessment and management. The endocrinologist can also advise about the likely need for endocrine replacement at puberty and, in adulthood, explain and monitor growth and fertility issues, and often has awareness of related medical issues in complex or multisystem cases (e.g., cardiac or renal anomalies). Adolescent endocrinologists play a key role in the induction of puberty, if needed, and adult endocrinologists with working experience of long-term administration and monitoring of hormone replacement therapies are essential for continuing long-term support as appropriate.

### A urologist/surgeon's view

The surgeon (paediatric surgeon or paediatric urologist) will always be a key member of the MDT, and may be the first point of referral, especially in those centres without an established MDT.

In specialist centres, the initial evaluation of the child with DSD should be performed in a joint manner by the surgeon and endocrinologist. Ideally, both of these individuals will have expertise in the management of such babies. The assessment will include a detailed history, identifying any points of relevance (family history, antenatal history and so on). The surgeon will need to carry out a detailed examination of the genitalia, focussing upon the presence and location of the gonads, the size and appearance of the phallus, the number and location of the urethral/vaginal orifices and the appearance of the labioscrotal folds and to confirm a normal situation of the anus. The findings of the examination should be discussed with the parents in a manner concordant with their level of understanding. In general terms, one should avoid the use of terminology that would favour one sex over another. For instance, using the term gonad rather than testis/ovary or phallus rather than penis/clitoris would be advised. Some parents may not be familiar with such medical terms and this would need to be ascertained from them at an early stage in the consultation.

One of the key questions asked by parents is in relation to the sex of their child (i.e., do they have a boy or a girl). Generally, the surgeon and endocrinologist can form an opinion about the appropriate sex of rearing at the initial consultation, particularly if preliminary investigations have already been carried out. Where uncertainty exists, a decision about sex of rearing should be deferred until investigation results are available and the MDT has been involved in the decision-making process. In such cases, detailed discussions about a possible sex of rearing and potential surgery required are best avoided at the initial meeting. Information should be given to the parents in a graded manner, to allow time for reflection and assimilation. This process may take a number of meetings with various members of the MDT, but key individuals such as the surgeon, endocrinologist and psychologist should always be present to maintain continuity. Often, parents will come with a body of knowledge ascertained from the referring centre or the Internet. In such instances, this information needs respectful consideration and clarification where incorrect or misleading information has been gained.

The surgeon will use the initial evaluation to start to build an image of the genitalia at presentation, consider if changes may occur in the early months of life and to determine what investigations are likely to be needed to help make the diagnosis and guide decisions about the sex of rearing (cystoscopy, vaginoscopy, laparoscopy and so on). Finally, the surgeon may want to consider the nature and number of surgical procedures that may be required to achieve a desired outcome and if the involvement of other surgical specialists (e.g., gynaecologist) is likely to be needed.

Ultimately, it is the surgeon's responsibility to undertake surgery for genital ambiguity if considered necessary, to restore function and achieve an acceptable cosmetic outcome. There are instances where surgery may not be required or desired by the parents or where surgery is best delayed until adolescence or adulthood. The latter approach is becoming increasingly popular and the effect of this paradigm shift will only become apparent in the future. Careful long-term follow-up is therefore of paramount importance.

Children raised male are likely to require at least two surgical procedures to reconstruct the penis in the first 2–3 years of life. In the majority of cases, the cosmetic and functional outcome is very good. In others where the penis is particularly small, a good outcome in terms of size may not be achievable. Some children raised male may require orchidopexy, or even gonadectomy where streak gonads are present. Müllerian structures of a significant size may also require removal, whilst smaller Müllerian remnants can be left *in situ* as usually they do not cause significant problems.

Children raised female may or may not require surgery in early childhood. Minor degrees of clitoral enlargement do not require surgical reduction and even when clitoral enlargement is significant, reduction surgery should only be carried out after the parents have been counselled about the advantages and risks of such a surgery. These discussions would require the involvement of a paediatric/adolescent gynaecologist from an early stage. In some children, the surgeon may recommend a vaginoplasty in the first 1–2 years of life, and indeed this is currently the standard practice in girls with a long urogenital sinus. This does not, however, obviate the need for further surgery in adolescence or early adulthood and parents should be made aware of this. It is entirely reasonable for those children with a relatively short urogenital sinus to have surgery deferred until adolescence or early adult life, thereby enabling the individual to be involved in the decision-making process.

Finally, the surgeon may be required to surgically remove gonads to protect the individual from gonadal malignancy. The timing of such surgery depends upon the nature of the DSD and the relative reported risk of malignancy.^1,11^ Decisions of an irreversible nature should be evidence based as much as possible and made in conjunction with the MDT.

### A gynaecologist's view

The role of the gynaecologist in the clinical management of a neonate or infant with a DSD is initially advisory only. A gynaecologist is not usually involved in the practical care of the small child but can offer information on long-term aspects such as sexual function and fertility. Parents may find it useful to meet and discuss potential adolescent and adult issues prior to taking a decision about genital surgery.

However, it is essential that a gynaecologist is involved in the care of children with a known DSD as they approach puberty and also in the care of newly diagnosed adolescent patients. Patients with a uterus will need a vaginal assessment at the onset of puberty to confirm there is a passageway for menstruation. This is usually performed under a brief general anaesthetic. If vaginal stenosis is present, then surgical reconstruction may be required. The examination may need to be repeated later in adolescence to ensure the vagina is adequate for both tampon use and sexual intercourse.

In those girls where the uterus is absent, vaginal examination can be deferred until later in adolescence and does not usually require an anaesthetic. If the vagina is short, vaginal dilation treatment is the treatment of choice and is successful in about 85% of patients.^12^ Those who are unsuccessful will require surgical reconstruction with a variety of methods such as the laparoscopic Vecchietti procedure or intestinal vaginoplasty, depending on the diagnosis and prior surgical intervention.

### A psychologist's view

The physical and psychological health needs for individuals with a DSD are closely interrelated. Medical management decisions in DSD are often based on promoting psychological adaptation and well-being rather than a physical health need *per se*. Even for individuals where decisions are made on the basis of physical considerations, the possible impact on psychological well-being needs to be taken into account. It is therefore essential for psychological well-being and adaptation to be seen as inseparable from medical management and for this reason psychological support and intervention are most effectively provided as an integral part of MDT care. The importance of a psychologist, or similar professional, in the management of DSD is not about pathologising DSD, but rather is recognition of the exceptional personal and social challenges that a diagnosis of DSD presents.

Adequate psychological care requires highly specialist skills and knowledge and in general should not be undertaken by local services in isolation from the MDT. The experience of working with individuals and families with DSD is essential to appreciate the complexity and sensitivity of the issues that commonly arise. In addition, an understanding of sex development and experience of gender identity development and factors influencing sex-role behaviour and sexuality together with knowledge of the trajectory of a DSD across development are required.

Psychological input is best considered as a process and not an event and contact with psychology is both routine, or pre-emptive, and reactive as issues arise. Planned episodes of medical care may not coincide with an individual's needs at particular points in time and, therefore, ideally the family and affected individual will feel able to initiate contact as and when required.

Psychological support is not embodied in the psychologist alone; rather, the presence of a psychologist in the team represents recognition of the interrelating physical and psychological aspects of DSD, as well as being a resource for individuals and their families with a DSD. It has become increasingly apparent that the way in which DSD is managed by health professionals affects psychological outcomes. Integrated holistic management has evolved in response to patient feedback and outcome research that has shown the long-term psychological distress associated with – among other things – surgery without consent, medical photography, repeated genital examinations, uncontained disclosure or unhelpful phrasing of sensitive information.^13^

Well-integrated MDTs provide scope for roles to develop based on the individual skills of the team members. Whilst there are tasks that clearly require the particular skills of a psychologist, other roles may emerge. In practice, the psychologist is often in a good position to act as an advocate and point of contact for families and individuals with a DSD and coordinate contact with other members of the team. Teamwork and close communication between team members facilitate the appropriate timing of medical interventions based on the needs of the young person; for example, when information needs to be given to a young person before the introduction of sex hormones at puberty or in cases where a decision needs to be made in puberty about gender identity. In this case, a gonadotrophin-releasing hormone (GnRH) agonist may be used to suppress puberty and to provide time and space for the psychologist to work with the young person and their family to reach a decision prior to any surgical intervention.

As an optimum, contact with psychology is initiated at the time of first contact with the team and, as with all members of the team, a collaborative, trusting relationship that respects and acknowledges individual, cultural, social and personal circumstances is actively fostered. Whilst the care of the individual with a DSD is central and foremost, important decisions are often taken on their behalf early in life by their family in collaboration with the MDT and, therefore, psychological care needs to encompass the family unit. There are crucial times when psychology has a role to play in relation to both medical management and individual development, including decisions about sex of rearing, surgical intervention, introduction of sex hormones, information about diagnosis and decisions about sex re-assignment, as well as routine support and monitoring of social and emotional well-being. In addition, psychological input should be available for unforeseen issues, including, for example, parental or individual concerns about gender identity development, school-related problems and bullying.

There are specific tasks at different stages of development, which should be planned with families from an early stage. In particular, families may require considerable support from the MDT to feel confident to give information to their child about their diagnosis and personal history in relation to this condition. The pacing and timing of giving information and making decisions is ideally based on individual circumstances, together with information about the DSD. Whilst decisions should be informed by evidence when possible, most often there is no ‘right’ course of action that applies in all circumstances. Rather there are broad goals, which are best approached with flexibility on a case-by-case basis. With this in mind, families and individuals require clear information about DSD and, sometimes, intensive input to empower them to successfully address challenges as they arise.

#### Issues at birth

Uncertainty about sex of rearing creates considerable practical and emotional challenges for parents. There is often an intense desire to move from uncertainty to certainty and a focus on medical intervention with the hope that it will provide the solution. In cases where diagnostic tests and physical examinations are not decisive in decisions regarding sex of rearing, it is particularly important to involve the psychologist, whose role is to provide time and space for discussion. The process of sex assignment is not only about making a decision but also an opportunity to process what has happened and set out plans for the future. Traumatic memories of the birth, careless words by professionals, misinformation from the Internet and unresolved fears about what will happen at puberty often resonate and can cause considerable distress and damage when they re-emerge at a later stage.

Initial discussions most often cover practical issues such as what to tell family and friends and a review of what the family has understood thus far with the aim of identifying fears and misconceptions. For families to make informed decisions, it is helpful to describe the process of sex determination and the implications of any decision into adult life, including puberty and fertility. In addition, psychological factors, including the definitions and distinctions between gender identity, sex-role behaviour and sexual orientation, together with known outcomes should be shared. Team meetings with the family to address questions that arise in discussions with the psychologist regarding the DSD, and possible medical interventions, are invaluable to maintain consistent information and prevent confusion.

#### Information management

Facilitating disclosure of information about a DSD has emerged as a key role for psychologists in the MDT. Commonly, parents do not feel they have the required knowledge to explain a DSD to their child and fear that information may cause distress or precipitate a crisis. Therefore, early and ongoing discussions with parents are required to address fears they may have about the impact of information on their child and to plan what information might be given when. The diagnosis of a DSD in adolescence presents particular challenges and is commonly associated with both the need to give information and to make difficult decisions about medical intervention or sex assignment.

#### Sex reassignment

There are particular circumstances that are exceedingly challenging both for the individual concerned and their family. The issue of sex reassignment can present at various stages throughout life. Typically, in a paediatric setting, the question can arise either shortly after birth, for example, when a male-assigned baby is found to be a 46,XX infant with CAH, during childhood when parents or the child express uncertainty about gender identity or at puberty when male-type secondary sexual development occurs in a female. Invariably, the team psychologist will be asked to provide an assessment. There are a number of standardised questionnaires available to aid assessment. Gender identity development is a complex area that requires considerable expertise to assess and then appropriately advise families. The implications of a change in gender need careful evaluation and planning. For example, families commonly request a change of both school and location. Any change in sex assignment after the age of about 12–18 months should only be considered if the child itself is fully involved and initiates the discussion.

In conclusion, whilst certain aspects of the management of DSD clearly require input from a psychologist or similar professional, their actual role in the MDT evolves with the team and it is this dynamic integration of medical and psychological support that provides the best model of care.

### A biochemist's view

A number of biochemical tests will be needed in the diagnosis and management of a patient with a DSD. A clinical scientist with an interest in DSD can advise the interdisciplinary team on the interpretation of tests and ensure effective use of small biological samples. Knowledge of assay specificities and reference ranges is important. A negative test result can be spotted and, through dialogue with the clinicians, the next stage of investigations can be instigated through further test requests.

The priorities for hormone tests are different depending on the age of the patient and the clinical problem. The assays needed are summarised in Table 2. In addition to steroid hormones and their regulatory peptides (adrenocorticotropic hormone (ACTH), luteinising hormone (LH) and follicle stimulating hormone (FSH)), assays for anti-Müllerian hormone (AMH), Müllerian inhibiting substance (MIS) and inhibin B have become available to provide further information about testicular development.^14,15^ Many laboratories will be able to meet some of the requests by assays available internally, but in many cases samples will be referred to specialist centres. Hormone assays are performed these days usually on automated platforms using immunoassay with different detection systems such as chemiluminescence. These assays are not always the best tests for samples from young patients because of the potential for interference from foetal and placental hormones. Many laboratories do not have the appropriate paediatric reference ranges – an important example here is plasma testosterone concentrations in genetic (46,XY) males that change with postnatal age in the first year of life. Testosterone concentrations up to 10 nmol l^−1^ are seen at birth and from 3 weeks to 6 months of age, but concentrations can be less than 1 nmol l^−1^ at 1 week of age and from 6 months of age until the start of puberty.^16^ When testosterone production is naturally low, stimulation tests of the axis may be needed. Plasma renin activity and aldosterone concentrations need to be known in some of the conditions of DSD. Few laboratories have reference ranges in children but interpretation of the results is dependent on knowledge of the normal levels. For some of the DSD conditions associated with adrenal failure, there is a need for rapid turn-around times for hormone assays; DSD causes much anxiety within the family and this should be borne in mind. The most common DSD in a 46,XX individual is CAH due to 21-hydroxylase deficiency. The analysis of 17-hydroxyprogesterone (17-OHP) concentrations in blood is a valuable diagnostic test. Many laboratories offer this test but most will use a direct assay (antibody coated-tube, radioimmunoassay) not appropriate for paediatric investigations. There are considerable interferences from foetal steroids in the first few days of life, so most laboratories will not accept samples until the child is at least 3 days of age. Kits for 17-OHP usually have a working range only to around 60 nmol l^−1^. These methods are not targeted for the much higher concentrations seen in classical CAH and are aimed at detection of heterozygotes and non-classic forms of CAH. To get a better measure of the plasma 17-OHP concentration in an infant with classical CAH, it will be necessary to use lesser amounts of sample. Ideally, steroid should be extracted into an organic solvent before immunoassay to leave foetal zone-derived steroid sulphate in the sample matrix.^17^ Assays are available using extraction and mass spectrometric detection that are specific and accurate from the first day of life.^18–20^ Wherever possible, such assays should be used. An abnormal result for one hormone should be treated with caution; the defect of cytochrome P450 oxidoreductase deficiency, for instance, affects the activity of two enzymes and corticosterone and progesterone should be measured as well as 17-OHP.^21^

Many laboratories are now looking at mass spectrometric techniques for steroid assays and in the next few years, an improvement in assay quality is anticipated.^20,22^ There is much variation in the quality of these steroid assays because, in essence, the assays were developed ‘in-house’. There are in use different standards, stable isotope-labelled internal standards, extraction techniques, chromatography, ionisation and fragment focussing. Many laboratories have little experience of mass spectrometry. Problems are encountered from matrix interference and skill is needed to spot these problems.^23^ Reference ranges will have to be established for children using new methods because results are lower than with current, less specific methods. The simultaneous analysis of several steroids has been achieved in a few specialised laboratories.

If the plasma 17-OHP is normal, then other steroid measurements will be needed (Table 2). In view of the range of diagnostic possibilities, a urinary steroid profile is an extremely useful test of adrenal function.^24,25^ Some of the plasma assays are not readily available (e.g., corticosterone). In general, androgens from the adrenal cortex are produced in very much greater amounts than from the gonads and abnormalities of testicular androgens are not apparent in the analysis of the metabolites in the urine. A defect of 5-α reductase is an exception through analysis of 5α- and 5β-reduced steroid ratios of androgens and cortisol metabolites. The urinary steroid profile test uses gas chromatography (GC) to separate the steroids in an extract of urine and the pattern over the time of the analysis indicates where steroids are deficient or produced in excess. The identification of steroids is improved when the output of the GC is a mass spectrometer. Each peak of steroid eluting from the GC has a characteristic molecular weight and fragmentation pattern. All of the adrenal genetic enzyme disorders can be identified in one analysis. A urine sample should, however, not be collected in the first 3 days because during that period of time there are many placental steroids in the urine of the child and clearance of the metabolites of the substrates for defective enzymes is relatively lower than in later samples. The laboratory needs experienced scientists to interpret the data and offer advice to clinicians. Detailed reference ranges for age and gender are needed because of changes throughout childhood.

The adrenal enzyme DSD disorders are amenable to genetic analysis, either as a clinical service or on a research basis.^26^ Other genetic analyses may be needed for androgen, LH and AMH receptors and other genes involved in developmental pathways (e.g., steroidogenic factor-1), under the guidance of clinical/molecular geneticists (see below).^27^

### A clinical/molecular geneticist's view

Clinical and molecular geneticists have several important roles to play as part of the DSD MDT.

Laboratory services are likely to be involved in the early stages of investigation by performing rapid fluorescence in-situ hydridisation (FISH) analysis of lymphocytes for the presence of X chromosomal and Y chromosomal material (e.g., using a probe directed at SRY). Close liaison with the laboratory is necessary, therefore, to inform them that this is an urgent rather than a routine sample, and to have the results interpreted in light of the clinical findings (e.g., ambiguous genitalia). Results are usually obtained within 48 h and provide a very important early component of the diagnostic pathway. A full G-banded karyotype should be checked when available to confirm initial findings and to ensure no significant chromosomal rearrangements, deletions or insertions or mosaicism is present. In some centres, initial rapid ‘karyotype’ analysis is performed by QFPCR of DNA. In complex cases, array comparative genomic hybridisation (CGH) or single-nucleotide polymorphism (SNP)/copy number variant (CNV) analysis may also be performed looking for submicroscopic deletions or duplications that might be relevant.

The clinical geneticist can provide valuable input for the diagnosis of many potential syndromic associations of DSD and in assessing dysmorphic or variant phenotypic features.^28^ Such features may be subtle, especially in the newborn. Reference to specialist literature, dysmorphology databases or clinical genetics review meetings might be needed, especially for unusual collections of features. The geneticist can also provide input into known single gene causes of DSD and can give guidance as to where genetic testing might be available, either as a clinical service or as part of an approved research protocol.^29^ Close interaction with academic laboratories may be necessary if detailed *in vitro* studies of protein function need to be performed to understand the effects of any change in more detail.

Finally, clinical genetics has a very important role to play in counselling individuals and/or their families about likely long-term aspects of specific conditions; inheritance patterns and the likelihood of other family members being affected or be carriers; and the advantages and risks of prenatal analysis and possible prenatal treatment options for conditions such as 21-hydroxylase deficiency. Professional training and experience in counselling is important.

### An ethicist's view

Ethics is often described as a set of rules or principles that distinguish between right and wrong; it is essentially about moral values rather then facts. When there appears to be no single obvious and acceptable ‘right' way to do something, ethics provides a means of evaluating and choosing between different, often competing options.

Ethical conflicts arise when:

(1) application of clinical facts alone cannot determine what should be done;
(2) there is disagreement about the right course of action;
(3) application of moral principles conflict; and
(4) the law is ambivalent or silent.

Ethical conflicts require professionals to make value judgements to resolve them. Since individuals within MDTs derive their own moral values from a wide range of sources, for example, family upbringing, education, religious beliefs and professional codes, it may be difficult for them to agree on which set of values they should apply in individual cases. Ethical intervention may provide clarification in a way that is supportive, educational and advisory rather than prescriptive.

Any ethical involvement with the MDT meeting should be first concerned with obtaining appropriate clinical facts that are founded on the best available evidence. These include

(1) the nature of the condition;
(2) its clinical effect on children, young people and parents;
(3) the associated clinical and psychosocial co-morbidities and their likely short- and long-term consequences; and
(4) the ‘reversibility’ of treatment options (e.g., hormone suppression vs. gonadectomy; neonatal vs. adolescent surgery).

#### Best interests

Although clinicians have a duty to act in the best interests of their patients^30^,objective standards of best interests for children may be hard to define in isolation. In DSD, there may be tensions between the potentially conflicting obligations of respecting a child or young person's right to make informed voluntary self-determined choices (autonomy) and providing treatment for the child that carries more benefit than harm. Moreover, any consideration of a child's best interests needs to include the obligation to respect the family and parent–child relationships.^31,32^

Traditional guidance for the timing and nature of therapeutic interventions in DSD may pay insufficient respect to the growing autonomy of adolescents and be overly paternalistic in its impact, or may restrict future life choices. The adverse effects of failing to respect the developing autonomy of adolescents are increasingly acknowledged.^33,34^

#### Dispute resolution

The structured format of the MDT meeting may be a useful forum for dispute resolution, provided if it has access to appropriate ethical expertise and knowledge of appropriate professional guidance and protocols as well as relevant legal principles.

Good ethical decision making requires that the process of decision making be as transparent, inclusive, reasonable and as accountable as possible. A practical approach to ethical decision making in facilitated MDT meetings with ethical input is outlined in Table 3.^35^

### Other members

Other health-care professionals, who may be involved in the DSD MDT, include specialists in radiology/imaging and histopathology (Fig. 1). Their attendance at meetings to discuss specific cases can be invaluable. Specialists in reproductive medicine may contribute to discussions about complex ethical and practical fertility issues and nurse specialists are in an ideal position to coordinate the team and provide a link with families. Academic input can be important if there are individuals associated with the team who have research interests in psychology, surgery, genetics or endocrinology.

The input of the local team can be extremely valuable. Parents often express their concerns, preconceptions and desires at an early stage but these might get overlooked in the diagnostic process; the local team may have insight into these issues. The primary-care doctor/general practitioner is often poorly informed about events, but may be an important source of support for the family and might reflect upon the situation in a less medicalised environment. Finally, local community or religious leaders can be very important in providing guidance in relation to personal religious doctrine, as well as providing ongoing support over time.

## The MDT meeting

The MDT meeting is an important forum during which the members of the team can gather and discuss relevant cases. The exact structure and organisation of the meeting will vary between centres, but in London we hold an MDT meeting to discuss newborn/paediatric cases once a month and a separate MDT meeting to discuss transition/adolescent/adult cases once every 2 or 3 months. If there is an urgent need for key members of the team to meet at other times, then this is arranged on an *ad hoc* basis, and discussions between team members occur on an ongoing basis throughout the month. The main MDT meeting is held in a room separate from the consultation room. This enables a broad spectrum of professionals to be able to give an opinion, without the family becoming overwhelmed or having their privacy invaded. However, the family should be aware of the structure of the meeting and not be made to feel excluded.

The MDT meeting provides an opportunity to

(1) review the clinical, cytogenetic, biochemical/endocrine, surgical and psychological findings for challenging cases where a sex assignment is still pending and to discuss the team's views or need for further investigation with the parents at the time;
(2) review recent data on cases where sex assignment has been made, but to obtain the team's opinion on diagnosis, management and counselling;
(3) review any new or updated information available on cases that have been discussed in the past (e.g., results of endocrine tests, findings at examination under anaesthesia (EUA), histology or feedback from psychologists following further discussions with the family);
(4) plan any specific investigations for children who may be coming into hospital soon (e.g., obtaining specific samples at surgery or liaising with histopathology/cytogenetics about specific tests); and
(5) have detailed discussions involving ethicists and other specialists (e.g., reproductive medicine) when key questions arise in relation to a complex case (e.g., sex reassignment and cryopreservation).

The MDT meeting brings together a large number of team members, as well as doctors, pathologists and psychologists in training, experts in key areas from other centres and the local team. It is essential to know everyone's identity, and that they have undergone approval to attend. A teaching session or journal review is often incorporated into the meeting, which forms part of ongoing education. An overview of the range of cases observed or discussed at the Great Ormond Street Hospital MDT meeting over 26 months (24 meetings) is shown in Table 4.

### Structure of the meeting

The MDT meeting at Great Ormond Street Hospital has been running for almost 20 years and the adolescent meeting for almost 10 years. During this time, the meeting has undergone various changes in format, which have been influenced by changes in the needs of parents and the team as well as changes in the environment. It is important that the discussion room is sufficiently soundproofed to avoid personal details being heard outside by others, and that any presentations or sensitive images should not be seen outside the venue (e.g., blinds drawn). It is important to let any parents attending the clinic know the structure of the clinic, likely time course and who they are likely to meet. Having some familiar faces is always reassuring, which is why the involvement of the local team is important if it is the family's first visit to the specialist centre.

### Organisation

Originally, the DSD clinic was held in a single large room with a video link to a smaller consulting room. This approach allowed the team to discuss details initially; then, only those key members involved in the case would need to have direct contact with the baby and family. With the parents' consent, the video link allowed the urologists' examination to be seen by the rest of the team without a large number of people being present. One disadvantage of this approach, however, was that only limited time was available to spend with the family; therefore, in recent years, we have adopted a largely discussion-based approach to the clinic and try to see families at the start or at the end of the main meeting. We usually see one or two families each meeting and are able to discuss three to five other cases. The normal length of the meeting is around 2.5–3 h, including a teaching session of 45 min. Increasingly complex cases have been reviewed in recent years and cases are often discussed on multiple occasions.

### Presentation, images, examination and governance

Case histories and data are usually presented in PowerPoint format from a secure hospital server. Unnecessary personal details or speculative comments should be avoided and biochemical and karyotype data should be double-checked with the original source beforehand. A copy of the presentation is usually put into the clinical notes as part of the medical record and electronic copies kept on a secure server in keeping with local data-protection policies. In some countries, individuals have the right to access all data held that concern them. This is in keeping with our policies of encouraging transparency and disclosure in relation to DSD. Other standard guidelines for medical confidentiality should be adhered to.^36^

Photographic or video images can be useful for the MDT to review, and can also be an important record for individuals and their parents later in life. Still photography should be viewed with caution as, for example, corporal size or consistency might not be apparent in a baby with suprapubic fat. Concurrent documentation from an experienced examiner (ideally an urologist) to accompany the photograph is needed. Video imaging of examination can be useful in some cases, and videos taken during surgical laparoscopy can be invaluable for reviewing internal structures. Any photography of external structures should be undertaken with full parental consent and ideally, only during anaesthesia in the older child. Images should be stored securely. Similarly, the examination should only be conducted in an appropriate environment and undertaken by the relevant team member. Genital examination should be limited to the minimal necessary in the older child and any detailed or invasive examination that might be necessary undertaken under short general anaesthetic.

### Discussions, documentation and databases

One of the most important features of the MDT meeting is to have an informed and balanced discussion of cases with the child's well-being as the central focus. Every case should be viewed on an individual basis, as social and psychological perceptions vary between families, and a spectrum of phenotypes can occur even for the same diagnosis. A literature review before the meeting is needed for rare conditions or unusual findings and, as far as possible, evidence-based approaches to decision making should be adopted.

The MDT meeting offers a unique forum for interactive discussion, review and contemplation. The MDT ‘consensus’ can give invaluable support to those key team members dealing directly with the families, can allow balanced review of data/diagnosis and can help to formulate a joint plan for future investigation and management. It is also important to question decisions and opinions by offering an opposing view. This can ensure that all options are well thought out, that the outcome of the discussions truly reflects consensus opinion and that advocacy for the child is provided within the context of the family. Sometimes, discussion with an ethicist or ethics committee is needed to try to resolve conflict of opinion within the team, or when decisions are extremely difficult (e.g., discordance between the parents' views and the recommendation of the MDT; see above).

Documenting the results of the discussions and management plan in the medical records and communicating these with the parents and relevant health-care professionals are important. It is often easy to overlook local services, who may offer invaluable support to the family. However, sensitive information should be disseminated on a ‘need to know’ basis, and ideally with the family's consent.

Local databases can be very useful for helping organise data on individuals being discussed at the MDT meeting and coordinating future plans. As noted above, any such databases should comply with local data-protection policies. Audit of local services might be useful and seeking feedback from parents, children/young people and adults attending the meetings and clinics is important to try to improve the ‘patient journey’. With changes in policy and practice, it is likely that an MDT meeting such as this will continue to evolve in the future.

## Follow-up in childhood

It is essential that all infants are followed up both in the short and long term to be able to monitor growth, assess the outcomes of surgery and to facilitate transition at the appropriate time.

During this process, it is important not to ‘medicalise’ the follow-up protocol for the child/young person, and we tend to minimise physical and, in particular, genital inspection.

It is important that there is a key physician and surgeon involved who will ideally follow the child through to adolescence with appropriate input from psychology. The psychologist will be closely involved in deciding the nature and timing of disclosure to the child/young person and will help in the planning of puberty and transition to the adolescent DSD service.

Facilitating the collection of long-term outcome data is essential to be able to provide feedback and positively influence early management decisions such as the need for, and timing of, genital surgery.

## Transition

There is little specific information on transition from paediatric to adult services for children with DSD. However, the general principles of transition are the same as for any chronic medical condition. Many of these are covered in the documents produced by the Department of Health.^37,38^ Generic issues include accessibility and co-location where possible of services, staff training, confidentiality and consent and health promotion. Young patients need to be encouraged to engage with services themselves and take a greater role in planning their own treatments. Adolescence is a time of uncertainty for all young people with the development of sexual identity and worries about relationships. These concerns are likely to be much greater in the presence of the diagnosis of a DSD. Understanding and coming to terms with an unusual karyotype or the requirement for further reconstructive genital surgery is a daunting prospect and specialised psychological input is crucial.

It would seem logical that the model of multidisciplinary care is just as appropriate for adolescents as it is for children. The involvement of both paediatric and adult clinicians can allow for careful transfer of information and strategic planning for the future. Specific milestones during transition are disclosure of diagnosis and genital assessment. The timing of disclosure varies hugely between patients and may be complete early in adolescence or not until the late teenage years. Either may be appropriate, but it is essential that there is close communication between clinicians involved in the progress.

Girls usually require vaginal assessment at some stage during adolescence. In the presence of a uterus, assessment may be required to confirm there is a passageway before menstruation starts. This is usually done under a brief general anaesthetic. In the absence of a uterus, the vaginal examination can be deferred until the girl is contemplating sexual activity and is usually performed in the outpatient clinic.

If vaginal dilation is required, success rates are good but are directly related to compliance. Support from a psychologist and supervision from a dedicated clinical nurse specialist are important. This usually provides an opportunity to explore worries about current and potential sexual relationships.

## Long-term outcome data

There is a general paucity of long-term outcome data for this group of patients as a whole, both male and female.^10^ Many adults with DSD have been lost to follow-up and historically this may, at least in part, have been due to insensitive or inappropriate care by clinicians. Delayed or even non-disclosure of the diagnosis was standard medical practice until relatively recently.^13^ As a result, it has often been difficult (both practically and ethically) to recruit into follow-up studies older patients who may not be aware of their karyotype. In addition, the original diagnoses may be incomplete or even incorrect.^39^

More recently, however, the advent of well-organised and better-informed patient peer-support groups, together with easy Internet access, has led to increasing numbers of older adult patients seeking information on every aspect of their condition. This should in turn increase our store of knowledge when advising younger patients, particularly as they approach adolescence.

It is essential that we plan a seamless pathway of transition, from paediatric through adolescent and into adult services. This will not only serve to optimise the care of individuals with DSD, but it will also ensure that both short- and long-term outcome data are collected and translated into better future management strategies.

## Summary

We believe that a holistic, multidisciplinary approach is mandatory for patients presenting with DSD. Input from a variety of health professionals facilitates rapid and effective assessment and investigation, especially if there is any doubt as to the correct sex of rearing. Early psychological input provides the families with immediate support and coping strategies. The development of audit and research is also facilitated through the collaboration of the various health professionals involved with these patients. A recent survey of key paediatric endocrinology centres in Europe reported an MDT dealing with DSD was established in the majority of those centres that responded.^40^ Whilst this is encouraging, the experience and training of team members may be variable and it is likely that services for young people and adults with DSD are less well established. The facilitation of adolescent transition and long-term follow-up by adult focussed MDTs will help to improve the future management of these conditions with a view to optimising the provision of health services for DSD and related conditions across the life span. Appropriate training, education and funding is needed if this goal is to be achieved.

**Practice points**

• Individuals with DSD should have access to an MDT with experience in the diagnosis and management of these conditions.
• An experienced psychologist plays a central role in the management of DSD across the life span.
• Although achieving an early diagnosis can help with management decisions and counselling, an individualised approach is needed in every case.
• Proper transition of young people to adolescent and adult services is essential, as is the establishment of adult DSD services based on an MDT model.

**Research agenda**

• Better understanding of the basic mechanisms of sex development is needed to improve diagnosis and to evaluate the biological effects of specific genes, proteins, hormones and environmental or epigenetic events.
• Improved biomarkers, imaging technologies and psychological tools are needed to assess gonadal function, androgen exposure/action, early tumour risk, reproductive potential and gender identity within the MDT setting.
• Further research is needed into optimal disclosure strategies and how best to transition care for young people with DSD into adult services.
• Good-quality long-term outcome studies are needed to evaluate the advantages and risks of intervention (surgical, psychological and hormonal) and to optimise strategies for tumour-risk management, fertility potential, sexual function, hormone replacement, bone health and quality of life.
• Better strategies are needed to educate health-care practitioners, patients and families and the general public about sex development, reproductive function and DSD.

## Figures and Tables

**Table 1:** An overview of different presentations associated with DSD at different ages.

Presentation	Prenatal	Birth	Infant	Child	Adolescent	Adult
*Prevalent*						
Ambiguous genitalia	(+)	+	(+)			
Amenorrhea (+/− pubertal development)					+	

*Less prevalent*						
Karyotype/phenotype discordance	+	+				
Bilateral undescended testes		+	+			
Hypospadias/small penis	(+)	+	+	+		
Adrenal failure		+	+	+		
Inguinal herniae in a girl		+	+	+		
Premature sex development			+	+		
Hypertension			+	+	+	+
Other features (e.g. fits, Wilms' tumor)		+	+	+	+	
Androgenization in puberty					+	
Gonadal tumour				(+)	+	+
Infertility					(+)	+

**Table 2:** Laboratory services for the initial investigation of DSD.

	Plasma (essential)	If first plasma tests not fully informative	Urine
46,XX DSD - newborn	17-OHP, cortisol, androstenedione, ACTH. Electrolytes. Synacthen test. In some cases will need plasma renin activity (concentration) and aldosterone concentration.	DHAS, 11-deoxycortisol	Steroid profile very effective for adrenal disorders. Characteristic patterns for defects of CYP21A2, HSD3B2, CYP11B1, CYP17, and POR. Very low levels of steroids are seen with defects of CYP11A1and StAR.
46,XY DSD - newborn	Plasma testosterone, DHAS, LH, FSH. AMH, inhibin B (ACTH, Synacthen test)*	Plasma androstenedione, testosterone, DHT (before and after hCG).	Steroid profile can exclude 17-hydroxylase and 3β-hydroxysteroid deficiencies but not helpful for testicular defects except 5α-reductase deficiency.

DHAS - dehydroepiandrosterone sulphate. DHT - 5-alpha dihydrotestosterone. * Consider tests of adrenal function in 46,XY DSD.

**Table 3:** A practical approach to ethical decision making (adapted from UKCEN materials^35^).

MDT meetings with ethical input should address the following Ascertain relevant clinical & psychosocial facts Separate facts from values of those involved- professionals and family Define an appropriate decision-making process e.g by determining who has responsibility? Who's involved in the process? What is the status of each? What is the time frame in which decision needs to be made? List options for action and their morally significant features e.g best interests, fairness Ascertain what legal or professional guidance exists Identify moral arguments for and against the options listed Choose an appropriate treatment option Identify the strongest ethical argument against its use, can it be rebuffed? Why? Make a choice Review outcomes and learn from experience

**Table 4:** Overview of the Great Ormond Street Hospital DSD meeting over approximately two years.

Sex Chromosome DSD (*n* = 13)	46,XY DSD (*n* = 65)	46,XX DSD (*n* = 23)
**A: 47,XXY** (Klinefelter Syndrome & variants) **(2)** **B: 45,X** (Turner Syndrome & variants) **(2)***(both Y fragment)* **C: 45,X/46,XY mosaicism** (mixed gonadal dysgenesis & variants) **(8)** **D: 46,XX/46,XY** (chimerism/mosaicism) **(1)**	**A: Disorders of gonadal (testis) development** 1. Complete gonadal dysgenesis **(7)** 2. Partial gonadal dysgenesis **(8)** 3. Steroidogenic factor-1 **(2)**	**A: Disorders of gonadal (ovary) development** 1. Ovotesticular DSD **(1)** 2. Testicular DSD **(1)**
	**B: Disorders in androgen synthesis or action** 1. Disorders of androgen biosynthesis STAR **(1)** 17β-HSD III **(2)** 5α-reductase II **(6)** 2. Disorders of androgen action CAIS **(6)** “PAIS” **(5)**	**B: Androgen excess** 1. Fetal 21-hydroxylase **(8)** 11β-hydroxylase **(2)** 2. Fetoplacental 3. Maternal
	**C: Other** 1. Syndromic associations of male genital development **(11)** (e.g. chromosomal variants, skeletal, lung, skin, gastrointestinal, Cornelia de Lange, CHARGE) 2. Cloacal anomalies **(1)** 3. IUGR/preterm/hypospadias **(4)** 4. Persistent Müllerian duct syndrome **(3)** 5. Vanishing testis syndrome **(2)** 6. Isolated severe hypospadias **(4)** 7. Micropenis **(1)** 8. Bilateral undescended testes **(2)**	**C: Other** 1. Syndromic associations (e.g. cloacal anomalies) **(1)** 2. Müllerian agenesis **(1)** 3. Clitoromegaly, possible clitoromegaly or clitoral variants **(8)** 4. Ovarian cysts **(1)**

Figures in bold (parentheses) show the current working diagnosis for individual cases seen or discussed during the DSD clinic over a 26 month period (24 multidisciplinary meetings). A total of 101 different cases were discussed and, in addition, 42 repeat discussions, reviews or updates took place. Therefore, 143 cases were considered during this time period (average: approximately 6 discussions per meeting). One case needed detailed input from ethicists. Of note, most cases of milder CAH or severe penoscrotal hypopadias were managed by the urology/endocrinology/psychology teams without referral to the joint MDT meeting.
